# Evaluation of *Leishmania donovani* Protein Disulfide Isomerase as a Potential Immunogenic Protein/Vaccine Candidate against Visceral Leishmaniasis

**DOI:** 10.1371/journal.pone.0035670

**Published:** 2012-04-23

**Authors:** Pramod Kumar Kushawaha, Reema Gupta, Chandra Dev Pati Tripathi, Shyam Sundar, Anuradha Dube

**Affiliations:** 1 Division of Parasitology, Central Drug Research Institute, Lucknow, India; 2 Department of Medicine, Institute of Medical Sciences, Banaras Hindu University, Varanasi, India; Instituto Butantan, Brazil

## Abstract

In Leishmania species, Protein disulfide isomerase (PDI) - a redox chaperone, is reported to be involved in its virulence and survival. This protein has also been identified, through proteomics, as a Th1 stimulatory protein in the soluble lysate of a clinical isolate of *Leishmania donovani* (LdPDI). In the present study, the molecular characterization of LdPDI was carried out and the immunogenicity of recombinant LdPDI (rLdPDI) was assessed by lymphocyte proliferation assay (LTT), nitric oxide (NO) production, estimation of Th1 cytokines (IFN-γ and IL-12) as well as IL-10 in PBMCs of cured/endemic/infected Leishmania patients and cured *L. donovani* infected hamsters. A significantly higher proliferative response against rLdPDI as well as elevated levels of IFN-γ and IL-12 were observed. The level of IL-10 was found to be highly down regulated in response to rLdPDI. A significant increase in the level of NO production in stimulated hamster macrophages as well as IgG2 antibody and a low level of IgG1 in cured patient's serum was observed. Higher level of IgG2 antibody indicated its Th1 stimulatory potential. The efficacy of pcDNA-*LdPDI* construct was further evaluated for its prophylactic potential. Vaccination with this construct conferred remarkably good prophylactic efficacy (∼90%) and generated a robust cellular immune response with significant increases in the levels of iNOS transcript as well as TNF-α, IFN-γ and IL-12 cytokines. This was further supported by the high level of IgG2 antibody in vaccinated animals. The *in vitro* as well as *in vivo* results thus indicate that LdPDI may be exploited as a potential vaccine candidate against visceral Leishmaniasis (VL).

## Introduction

Visceral Leishmaniasis (VL) is the most severe systemic disease among the three main categories of leishmaniasis and affects 500,000 people every year [1], [2]. Moreover, VL has emerged as an opportunistic infection in HIV-1 infected patients in many parts of the world [3], [4], [5]. Currently, there is no effective vaccine against leishmaniasis and control of the disease is almost confined to chemotherapy. There are only a limited number of drugs available and each has its own disadvantages, as they require long-term administration periods and often induce serious side-effects due to their toxicity [6], [7]. In addition, increasing incidence of drug-resistant strains has hampered the control of the disease even by chemotherapy [8], [9], [10], [11]. Therefore, attention has now been shifted towards the development of effective vaccines. Although induction of lifelong protection against re-infection in individuals who recovered from the disease, demonstrates that a protective vaccine can be achieved, an effective vaccine against human leishmaniasis has yet to be discovered [12].

The outcome of the typical symptomatic clinical form of VL is critically influenced by the immune response developed by the host wherein the systemic infection, with spread of the parasites to the spleen, liver, lymph nodes, bone marrow and other organs, is accompanied by a high titre of circulating antibodies and a depression of Type 1 T-cell mediated immunity, with decreasing production of IFN-γ and IL-12 and a marked up-regulation of IL-4 and IL-10 [13], [14], [15], [16], [17], [18]. However, asymptomatic clinical form in VL endemic regions is often followed by protective immunity, where a predominant Type 1 T-cell response is observed [19]. These findings suggest that any intervention that helps the shift of the immune response from Th2 type toward Th1 type will have a major role in cure and prevention of VL. Therefore, strategies to immune-potentiate the Th1 arm of the immune response could be exploited for the development of potential vaccine candidates. Further, genetic immunization is a relatively new tool for achieving specific immune activation with several advantages such as expression of concerned genes nearest to its native form, induction of cellular immune response, persistent expression of desired antigen and induction of memory responses against the infectious disease [20]. Moreover, host cells take up coding plasmids, transcribe and translate the encoded gene, and produce proteins that stimulate an immune response when presented to the immune system in the context of self-MHC [21], [22], [23]. Notably, vaccination with plasmid DNA has been shown to induce protective immunity through both MHC class I and class II restricted T cell responses in a variety of infections [24], [25], [26]. Therefore, the plasmid DNA encoding specific antigen induced both CD4+ and CD8+ T cells, which is essential for protection against intracellular diseases that require cell mediated immunity like leishmaniasis [27]. However, DNA vaccines have made little progress in the field of VL. Vaccination with the ORFF gene induced both humoral and cellular immune responses against ORFF, which provided a significant level of protection against challenge with *L. donovani* in a mouse model [28]. Similarly, a DNA vaccine based on kinetoplastid membrane protein 11 (KMP11) provided protection to golden hamsters against both antimony-responsive and -resistant Leishmania strains developed [29]. In an attempt to identify new antigens to be used as vaccines, we focused on those proteins of *Leishmania* parasites which are Th1 stimulatory and supposed to be the effector molecules for defence mechanism against *Leishmania* infection. Protein disulfide isomerase (PDI) was identified through proteomics as one of the soluble leishmanial protein that induced a Th1 response in the PBMCs of *Leishmania* infected cured/endemic patients [30].

PDIs are essential enzymes that catalyze thiol-disulfide interchange, ensuring the proper folding and conformation of proteins, acting as co-receptors of cell reorganization, and preventing cell toxicity associated with ER stress and protein misfolding [31], [32]. PDIs may also be involved in the host mucosal immune system, inducing secretory IgA [33], [34]. Although much has been learned about PDIs in higher eukaryotes, limited information is available regarding PDIs in pathogens that are important for human infections. The protein has been reported to have role in *Leishmania* virulence and survival [35]. The characterization of these virulence factors obviously has important implications for the design of new drugs or vaccines against *Leishmania* parasites. The present study deals with the cloning, expression and purification as well as molecular characterization of LdPDI. The protein was further examined for the first time for its ability to 1) stimulate the immune responses in leishmania infected cured/endemic contact individuals' PBMCs, 2) modulate the immune response in cured hamsters infected with *L. donovani* and 3) protect the hamsters against *L. donovani* challenge when administered as DNA vaccine. The hamster (*Mesocricetus auratus*) has been proven to be the most appropriate experimental model as it largely reflects the clinic-pathological features of progressive human VL, including a relentless increase in visceral parasitic burden, cachexia, hepatosplenomegaly, pancytopenia, hypergammaglobulinemia and ultimately death. Of late, it has also been used extensively for vaccination studies [36], [37].

## Materials and Methods

### Animal

Laboratory-bred male golden hamsters (*Mesocricetus auratus*, 45–50 g) from the Institute's animal house facility were used as experimental host. They were housed in climatically controlled room and fed with standard rodent food pellet (Lipton India Ltd., Bombay) and water *ad libitum*. The usage of hamsters was approved by the Institute's Animal Ethical Committee (protocol number 150/09/Para/IAEC dated 23.10.2009).

### Parasites

*The L. donovani* clinical strain was procured from a patient admitted to the Kala-azar Medical Research Centre of the Institute of Medical Sciences, BHU, Varanasi and was cultured *in vitro* as described elsewhere [37]. Promastigotes were grown in RPMI – 1640 medium at 26°C (Sigma, USA) in 75 cm^2^ culture flasks (Nunc) [38]. The strain has also been maintained in hamsters through serial passage, i.e. from amastigote to amastigote [38].

### Preparation of soluble *L. donovani* promastigote antigen

Soluble *L. donovani* (SLD) promastigote antigen was prepared as per method described by Gupta *et al*. [30]. Briefly, log phase promastigotes (10^9^) were harvested from 3 to 4 days of culture and washed four times in cold phosphate-buffered saline (PBS) and resuspended in PBS containing protease inhibitors cocktail (Sigma, USA) and subjected to ultrasonication and centrifugation at 40,000×g for 30 min.The protein content of the supernatant was estimated [39] and stored at −70°C.

### Cloning, expression and purification of recombinant LdPDI (rLdPDI)

*L. donovani* genomic DNA was isolated from 10^8^ promastigotes, washed and suspended in NET buffer (10 mM Tris-HCl (pH 7.5), 100 mM NaCl, and 1 mM EDTA) and incubated with proteinase K (1 mg/ml; Invitrogen Life Technologies) and 0.5% SDS at 50°C for 4 h. Nucleic acids were extracted by phenol∶chloroform∶isoamyl alcohol extraction and ethanol precipitation. Genomic DNA was spooled and subjected to RNase (100 µg/ml) treatment. *LdPDI* gene was amplified using Taq Polymerase (Takara) lacking a 3′–5′ exonuclease activity. PCR was performed using *LdPDI* -specific primers (based on the *L. infantum* - PDI gene sequence): forward, 5-AAGCTTATGCAGCGCTCATTCCTTGC-3 and reverse, 5- GCGGCCGCCCTACAAATCTTCCTCTTCG-3 (*HindIII* and *NotI* site underlined) in a Thermocycler (Bio-Rad) under conditions at one cycle of 95°C for 2 min, 30 cycles of 95°C for 1 min, 50°C for 1 min., and 72°C for 1.30 min, and finally one cycle of 72°C for 10 min. Amplified PCR product was electrophoresed in agarose gel and eluted from the gel by Gen Elute Columns (Qiagen). Eluted product was ligated in pTZ57R/T (T/A) cloning vector (Fermentas) and transformed into competent DH5α cells. The transformants were screened for the presence of recombinant plasmids with the *LdPDI* gene-specific PCR under similar conditions as previously mentioned. Isolated positive clones were sequenced from Delhi University (New Delhi) and submitted to the National Center for Biotechnology Information http://www.ncbi.nlm.nih.gov/nuccore/EU723849 (accession no. EU723849). *LdPDI* was further subcloned at the *HindIII* and *NotI* site in bacterial expression vector- pET28a (Novagen) as well as mammalian expression vector pcDNA3 for expression under the control of a strong cytomegalovirus (CMV) promoter. The expression of recombinant LdPDI protein (rLdPDI) was checked in bacterial cells by transforming the pET - *LdPDI* construct in *Escherichia coli* Rosetta strain. The transformed cells were inoculated into 5-ml test tube Luria- Bertani medium (LB) and allowed to grow at 37°C in a shaker at 220 rpm. Cultures in logarithmic phase (at OD_600_ of ∼0.5–0.6) were induced for 3 h with 1.0 mM isopropyl-b-D-thiogalactopyranoside (IPTG) at 37°C. After induction, 1 ml cells were lysed in 100 µl sample buffer (50 m M Tris-HCl (pH 8), 10% glycerol, 10% SDS, and 0.05% bromophenol blue, with 100 mM DTT) and whole cell lysates (WCL) was analyzed by 12% SDS-PAGE (32). Uninduced control culture was analyzed in parallel.

For purification 200 mL of LB medium containing 34 µg/mL of chloramphenicol and 35 µg/mL kanamycin were inoculated with *E.coli*. rosetta strain transformed with pET - *LdPDI*, and grown at 37°C to an O.D._600_ of ∼0.6. Recombinant protein expression was induced by addition of 1 mM (IPTG,Sigma) and the culture was incubated for an additional 4 to 5 h. The rLdPDI was purified by affinity chromatography using Ni^2+^chelating resin to bind the His6-tag fusion peptide derived from the pET28a vector. The cell pellet was resuspended in 4 mL of lysis buffer (50 mM Tris-HCL (pH 8.0), 300 mM NaCl, and 20 mM imidazole,) containing 1∶200 dilution of protease cocktail inhibitor (Sigma) and 1% Triton X-100, incubated for 30 min on ice with 1 mg/mL lysozyme (Sigma), and the suspension was sonicated for 10×20 sec (with 30 s intervals between each pulse) on ice. The sonicated cells were centrifuged at 15,000 g for 30 min, and the supernatant was incubated at 4°C for 1 h with the 2 mL of Ni–NTA Superflow resin (Qiagen, Hilden, Germany) previously equilibrated with lysis buffer. After washing with buffer (50 mM Tris-HCL(pH 8.0), 300 mM NaCl and 1% Triton X-100), containing different concentrations of imidazole i.e. 20,50 and 100 mM, the purified rLdPDI was eluted with elution buffer (50 mM Tris-HCL, 100 mM NaCl, and 250 mM imidazole, pH 7.5). The eluted fractions were analyzed by 12% SDS–PAGE and the gels were stained with Coomassie brilliant blue R-250 (Sigma–Aldrich, St. Louis, USA). The protein content of the fractions was estimated by the Bradford method using bovine serum albumin (BSA) as standard. The presence of endotoxin was tested by QCL-1000® Chromogenic LAL kit (Lonza).

### Production of polyclonal antibodies against the recombinant Protein and Western blot analysis

The purified rLdPDI protein was used for raising antibodies in New Zealand white rabbit. Rabbit was first immunized using 50 µg of rLdPDI in Freund's complete adjuvant. After 15 days, the rabbit was given 3 booster doses of 25 µg rLdPDI each in incomplete Freund's adjuvant at 2-weeks interval and blood was collected for serum 8 days after the last immunization. For immunoblotting experiment, purified rLdPDI protein and SLD were resolved on 12% SDS–PAGE and transferred on to nitrocellulose membrane using a semi-dry blot apparatus (Amersham) [40]. After overnight blocking in 5% BSA, the membrane was incubated with antiserum to the rLdPDI protein at a dilution of 1∶5000 for 120 min at room temperature (RT). The membrane was washed three times with PBS containing 0.5% Tween 20 (PBS-T) and then incubated with goat anti-rabbit IgG HRP conjugate (Bangalore Genie) at a dilution of 1∶10,000 for1 h at room temperature. Blot was developed by using diaminobenzidine+imidazole +H_2_O_2_ (Sigma).

### Expression of pcDNA-*LdPDI* in mammalian cell line

The expression of pcDNA-*LdPDI* was further checked in mammalian cells by transfecting the pcDNA-*LdPDI* construct in Human Embryonic Kidney-293 T (HEK- 293T) cell line cells. This cell line was commercially obtained from American Type Culture Collection (ATCC). For transfection and vaccination studies, endotoxin- free plasmid DNA was isolated using an Endofree plasmid purification Maxi kit as per the manufacturer's protocol (Qiagen). For confirmation of the proteins expressed by the pcDNA-*LdPDI* construct, 2×10^5^ HEK - 293T cells were grown in four-chamber slides and transfected with the blank pcDNA3 plasmid and pcDNA-*LdPDI* construct, in serum-free DMEM (Life Technologies) using Lipofectamine 2000 (Invitrogen) according to the manufacturer's protocol. Expression was confirmed by RT-PCR and western blotting. The lysate of stably transfected HEK- 293T cells was prepared and subjected to SDS-PAGE. Thereafter, the protein bands were electrophoretically transferred to nitrocellulose membrane. To detect the expressed protein, a primary polyclonal antibody against rLdPDI was used at 1∶5000 dilution followed by 1∶10000 dilution of HRP-conjugated goat anti-rabbit IgG secondary antibody (Bangalore Genei, Bangalore, India).

### Patients and isolation of peripheral blood mononuclear cells (PBMCs)

The study groups for human samples were as follows:

[1] Seven treated cured patients (4 males and 3 females, age ranging from 7–40 years) from hyper-endemic areas of Bihar. All the patients had received complete course of amphotericin B and had recovered from VL. Samples were collected from 2 months to 1 year after the completion of treatment. Diagnosis was established in all cases by demonstration of parasites in splenic aspirates and found negative at the time of study.

[2] Five endemic household contacts (2 males and 3 females, age range-15 to 40 years) who neither showed clinical symptoms nor received any treatment for Kala-azar.

[3] Five infected patients (3 males and 2 females, age range- 5 to 40 years) showing clinical symptoms of Kala-azar.

[4] Five normal healthy donors (3 males and 2 females, age range 20–30 years) from non-endemic areas, without any history of leishmaniasis, served as negative control.

Heparinized venous blood (10 ml each) was collected from all the study subjects and peripheral blood mononuclear cells (PBMCs) were isolated from blood by Ficoll Hypaque density gradient centrifugation (Histopaque 1077, Sigma, USA) as described by Garg *et al*. [37]. A final suspension of 1×10^6^ cells/ml was made in complete RPMI medium (cRPMI) after determining cell viability by trypan blue staining method. These were used for various immunological assays.

### Treatment of *L. donovani* infected hamsters and isolation of mononuclear cells (lymph node cells)

Approximately 20 hamsters, infected with 10^7^ amastigotes intracardially, were assessed one month later for parasitic burden by splenic biopsy through a small incision in the upper left quarter of the abdomen and a small piece of splenic tissue was cut and dab smears were made on slides. The incised portion was stitched with nylon suturing thread. Following biopsy, an adequate amount of antibiotic powder (Neosporin) was applied on the stitched portion and finally sealed with tincture benzoin. In addition, neosporin sulphate (100 mg/kg of body weight) was also given orally the day before and the day after the biopsy for healing. The smears were fixed in methanol and stained with Giemsa stain and the number of amastigotes/1000 cell nuclei was counted. The animals harbouring >25–30 amastigotes/100 macrophage cell nuclei were then treated with antileishmanial drug-Miltefosine (Zentaris, Germany) at 40 mg/kg bodyweight daily for 5 days. The animals were reassessed for complete cure by splenic biopsy performed on day 30 post-treatment.

Mononuclear cells were separated from lymph nodes of cured, infected as well as normal hamsters following the protocol of Garg et al, [37] and a suspension of 10^6^ cells/ml was made in cRPMI. These cells were employed for lymphoproliferative assay and for the estimation of NO production.

### Assessment of prophylactic efficacy of pcDNA-*LdPDI* in hamsters to Leishmania challenge

Four experimental groups each having 12–15 hamsters were taken for the study. The hamsters of Group 1 was immunized intramuscularly (i.m.) with pcDNA-*LdPDI* (100 ug/100 ul PBS/animal) whereas groups 2 to 4 served as controls. The animals of group 2 were vaccinated with 100 µg of pcDNA3 (blank vector)/100 µl of PBS and that of group 3 served as unvaccinated but infected control. The animals of group 4 did not receive either pcDNA-*LdPDI* or *Leishmania* infection served as normal control. On day 7 and 14, two booster doses of 100 µg pcDNA-*LdPDI* were given i.m. to group 1.Twenty-one days after the first vaccination, all the vaccinated and unvaccinated control groups were challenged intracardially with 10^8^ late log phase promastigotes of *L. donovani*. The weight of body, spleen and liver (on necropsy) of hamsters of all of the experimental groups were assessed, on necropsy, at different time intervals, i.e., on days 0, 60, 90 and 120 p.c. The assessment of parasite burden was done in spleen, liver and bone marrow of vaccinated hamsters at different time intervals, i.e. on days 0, 45, 90, 120 post-challenge (p.c.). Peritoneal exudates cells, inguinal lymph nodes and blood were also collected at these time points to obtain cells and sera for evaluation of cellular and antibody responses as per protocols described above. The criterion for prophylactic efficacy was the assessment of parasite load as the number of amastigotes/1000 cell nuclei in each organ in comparison to the unvaccinated controls and the percentage inhibition (PI) was assessed in comparison to the unvaccinated control by following formula [41] PI = (No. of parasite count from infected control –No. of parasite from vaccinated group/No. of parasite count from infected control)×100. DTH was performed by injecting 50 µg/50 µl of SLD in PBS intradermally into one footpad and PBS alone into the other one of each of the vaccinated and unvaccinated controls. The response was evaluated 48 h later by measuring the difference in footpad swelling between the two with and without SLD for each animal [42].

### Immunological assay

#### Assessment of Lymphocyte proliferative responses (LTT) in cured/exposed patients and hamsters

Lymphocytes suspension (1×10^6^ cells/ml) of cured/exposed patients and normal, infected (30 days p.c.) and cured hamsters was cultured in 96-well flat bottom tissue culture plates (Nunc, Denmark). This assay was carried out as per protocol described by Garg et al. [37] with some modifications, wherein XTT (Roche diagnostics) was used instead of ^3^H thymidine. About 100 µl of predetermined concentration of mitogens, PHA (10 µg/ml Sigma, USA) for Patient's PBMCs, Con A (10 µg/ml, Sigma), for hamster's lymphocytes, as well as rLdPDI and SLD (10 µg/ml each) were added to the wells in triplicate. Wells without stimulants served as blank controls. Cultures were incubated at 37°C in a CO_2_ incubator with 5% CO_2_ for 3 days in the case of the mitogens, and for 5 days in the case of the antigens. Eighteen hours prior to termination of experiment, 50 µl of XTT was added to 100 µl of supernatants of each well and absorbance measured at 480 nm with 650 nm as reference wavelength.

#### Estimation of NO activity in macrophages of hamsters and cell lines

Isolated lymphocytes from all the three study groups of hamsters viz. Normal, infected (30 p.c.) and cured, were suspended in culture medium and plated at 10^5^ cells/well and stimulated for 3 days in case of mitogen (LPS) and 5 days in case of antigens (rLdPDI, SLD) at 10 µg/ml. The presence of NO was assessed using Greiss reagent(Sigma, U.S.A) in the culture supernatants of naive hamster peritoneal macrophages [37] after the exposure with supernatant of stimulated lymphocyte's. The supernatants (100 µl) collected from macrophage cultures 24 h after incubation was mixed with an equal volume of Greiss reagent (Sigma, USA) and left for 10 min at room temperature. The absorbance of the reaction was measured at 540 nm in an ELISA reader [43]. The nitrite concentration in the macrophages culture supernatant samples was extrapolated from the standard curve plotted with sodium nitrite.

#### Assessment of Cytokine levels- IFN-γ/IL-12/IL-10 in lymphocytes of cured/endemic patients

Culture of PBMCs (1×10^6^ cells/ml) from human patients was set up in 96-well culture plates and rLdPDI was added in triplicate wells at a concentration of 10 µg/ml. The level of IFN-γ, IL-12 and IL-10 was estimated by enzyme-linked immunosorbent assay (ELISA) (OptEIA set, Pharmingen) after 5 days of incubation with antigen using supernatants. The results were expressed as picograms (pg) of cytokine/ml, based on the standard curves of the respective cytokine provided in the kit. The lower detection limits for various cytokines were as follows: 4.7 pg/ml for IFN-γ, 7.8 pg/ml for IL-12p40, 7.0 pg/ml for IL-10.

#### Quantification of mRNA cytokines and inducible NO synthase (iNOS) in hamsters by Real time-PCR

qRT-PCR was performed to assess the expression of mRNAs for various cytokines and iNOS in splenic cells. Splenic tissues were taken from each of the 3 to 4 randomly chosen animals. Total RNA was isolated using Tri-reagent (Sigma-Aldrich) and quantified by using Gene-quant (Bio-Rad). One microgram of total RNA was used for the synthesis of cDNA using a first-strand cDNA synthesis kit (Fermentas). For qRT-PCR, primers were designed using Beacon Designer software (Bio-Rad) on the basis of cytokines and iNOS mRNA sequences available on PubMed [36] (Table 1). qRT-PCR was conducted as per the protocol described earlier [44]. Briefly, it was carried out with 12.5 µl of SYBR green PCR master mix (Bio-Rad), 1 µg of cDNA, and primers at a final concentration of 300 nM in a final volume of 25 µl. PCR was conducted under the following conditions: initial denaturation at 95°C for 2 min followed by 40 cycles, each consisting of denaturation at 95°C for 30 s, annealing at 55°C for 40 s, and extension at 72°C for 40 s per cycle using the iQ5 multicolor real-time PCR system (Bio-Rad). cDNAs from infected hamsters were used as “comparator samples” for quantification of those corresponding to test samples. All quantifications were normalized to the housekeeping gene, HPRT. A no-template control cDNA was included to eliminate contaminations or nonspecific reactions. The cycle threshold (CT) value was defined as the number of PCR cycles required for the fluorescence signal to exceed the detection threshold value (background noise). Differences in gene expression were calculated by the comparative CT method [44]. This method compares test samples to a comparator sample and uses results obtained with a uniformly expressed control gene (HPRT) to correct for differences in the amounts of RNA present in the two samples being compared to generate a ΔCT value. Results are expressed as the degrees of difference between ΔCT values of test and comparator samples.

**Table 1:** Sequences of forward and reverse primers of hamster cytokines used for quantitative real time -PCR.

S.N.	Primer	Primer Sequence
1	HPRT	Forward 5′ GATAGATCCACTCCCATAACTG 3′
		Reverse 5′ TACCTTCAACAATCAAGACATTC 3′
2	TNF-α	Forward 5′ TTCTCCTTCCTGCTTGTG 3′
		Reverse 5′ CTGAGTGTGAGTGTCTGG 3′
3	IFN-γ	Forward 5′ GCTTAGATGTCGTGAATGG 3′
		Reverse 5′ GCTGCTGTTGAAGAAGTTAG 3′
4	IL-12	Forward 5′ TATGTTGTAGAGGTGGACTG 3′
		Reverse 5′ TTGTGGCAGGTGTATTGG 3′
5	TGF-β	Forward 5′ ACGGAGAAGAACTGCTGTG 3′
		Reverse 5′ GGTTGTGTTGGTTGTAGAGG 3′
6	IL-4	Forward 5′ GCCATCCTGCTCTGCCTTC 3′
		Reverse 5′ TCCGTGGAGTTCTTCCTTGC 3′
7	IL-10	Forward 5′ TGCCAAACCTTATCAGAAATG3′
		Reverse 5′ AGTTATCCTTCACCTGTTCC 3′
8	iNOS	Forward 5′ CGACGGCACCATCAGAGG 3′
		Reverse 5′AGGATCAGAGGCAGCACATC 3′

#### Measurement of antibody response in hamsters and Cured patients

The level of IgG antibody and its isotypes in sera samples of hamsters of different experimental groups as well as in cured patient serum was measured as per protocol by Samant et al. [44]. Briefly, 96-well ELISA plates (Nunc) were coated with rLdPDI/SLD (0.2 µg/100 µl/well) overnight at 4°C and blocked with 1.5% BSA at room temperature for 1 h. Sera was used at a dilution of 1/100 for IgG, IgG1, and IgG2 and kept for 2 h at room temperature. Biotin-conjugated mouse anti-Armenian and Syrian hamster IgG, IgG1 and biotinylated anti-Syrian hamster IgG2 (BD Pharmingen) for hamster and mouse anti human IgG, IgG1 and IgG2 in case of human were added for 1 h at room temperature at 1/1000 dilutions and were further incubated with streptavidin-conjugated peroxidase at 1/1000 (BD Pharmingen) for 1 h. Finally, the substrate O-phenylenediamine dihydrochloride (Sigma-Aldrich) was added and the plate was read at 492 nm.

#### Post-challenge survival and Statistical analysis

Survival of hamsters belonging to group 4 was checked until day 180 p.c. in comparison to the normal hamsters. Animals in all of the groups were given proper care and were observed for their physical conditions until their survival period. Survivals of individual hamsters were recorded and mean survival period was calculated. Results were expressed as mean±S.D. In each experiment 6–8 animals were used in each group. Two sets of experiments were performed and the results were analyzed by one-way ANOVA test followed by Dunnet's or Tukey's post which ever appropriate at each case using Prism Graphpad software program. The upper level of significance was chosen as p<0.001 (highly significant).

#### Ethics Statement

The study and the protocol was approved by the Ethics committee of the Kala-azar Medical Research Centre, Muzaffarpur (Protocol # EC-KAMRC/Vaccine/VL/2007-01) and CDRI and all patients provided a written consent before enrolment to this study. Experiments on the animals (hamsters) were performed following the approval of the protocol and the guidelines of Institutional Animal Ethics Committee (IAEC) of the CDRI. The approval reference number is 150/09/Para/IAEC dated 23.10.2009.

## Results

### *LdPDI* was cloned, sequenced, expressed in *E. coli* Rosetta strain and HEK cell line

**Figure 1:**
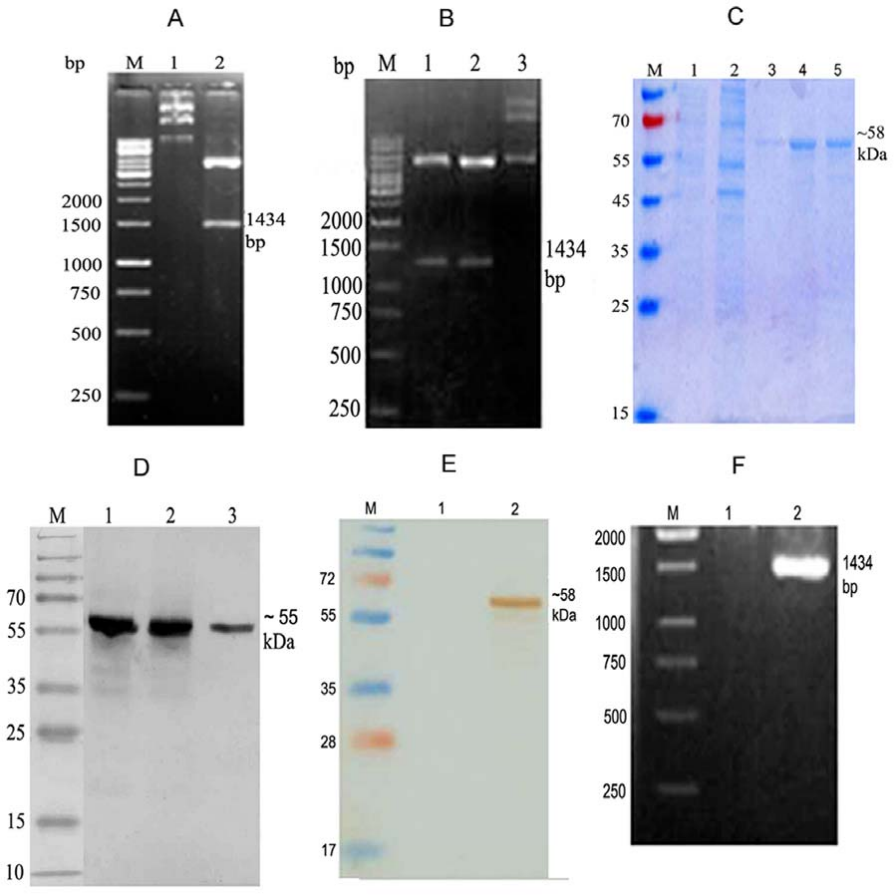
Molecular characterization of *Leishmania donovani* Protein disulfide isomerise (*LdPDI*). (A) Clone confirmation of *LdPDI* in TA and pET28a vector. M: 1 kb molecular mass marker; Lane 1: undigested plasmid, Lane 2: *Hind III* and *NotI* – digested TA- *LdPDI.* (B) Clone confirmation in pET28a. M: 1 kb molecular mass marker; Lane 1: *Hind III* and *NotI* – digested pET- *LdPDI*, Lane 2: Undigested pET- *LdPDI.* (C) Purification and elution of rLdPDI at 250 mM of imidazole concentration. M- Molecular wt. Markers, Lane 1&2: wash fraction, Lane 3 & 4: eluted protein. (D) Western blot with whole cell lysate and SLD of *L. donovani*, M- Molecular wt. markers Lane 1: purified protein, Lane 2&3 WCL and SLD respectively of *L. donovani.* (E) Western blot of transfected and control HEK cells, M- Molecular wt. Markers Lane 1: pcDNA control, Lane 2: transfected with pcDNA-*LdPDI*. (F) Confirmation of expression in HEK cell line by RT-PCR. M- 1 kb molecular mass marker Lane 1: pcDNA control, Lane 2: pcDNA-*LdPDI* transfected.

The *LdPDI* gene of *L. donovani* was successfully amplified, cloned in T/A vector (Fig. 1A) and sequenced, which was 99% homologous with *L.infantum* PDI (Table 2). It was further cloned in bacterial expression vector pET28a (Fig. 1B), purified and eluted at 250 mM imidazole concentration. The size of the eluted rLdPDIs was ∼58 kDa (Fig. 1C). Western Blot analysis of *L. donovani* promastigotes lysates was performed with the polyclonal anti- rLdPDI antibody which detected one dominant protein band of ∼55 kDa (Fig. 1D). Because the expression of recombinant proteins by mammalian cells transfected with bacterial plasmid DNA is a critical condition for the stimulation of the immune system, the expression of the rLdPDI protein was initially assessed in HEK-293T cells transfected with the pcDNA-*LdPDI* construct. Transfected cells were cultured for 2–3 days and washed, and expression of the recombinant proteins was assessed by SDS-PAGE and Western blot analyses. Cells transfected with pcDNA-*LdPDI*, expressed high levels of the recombinant protein as detected by Western blotting (Fig. 1E) and was further confirmed by RT-PCR (Fig. 1F)

**Table 2:** Homology of *LdPDI* of *L. donovani* with other Leishmania species.

Species	Homology (%)
*Leishmania infantum*	99
*Leishmania major*	94
*Leishmania braziliensis*	84

### rLdPDI induced strong lymphoproliferative and NO responses in normal/infected/cured hamsters

**Figure 2:**
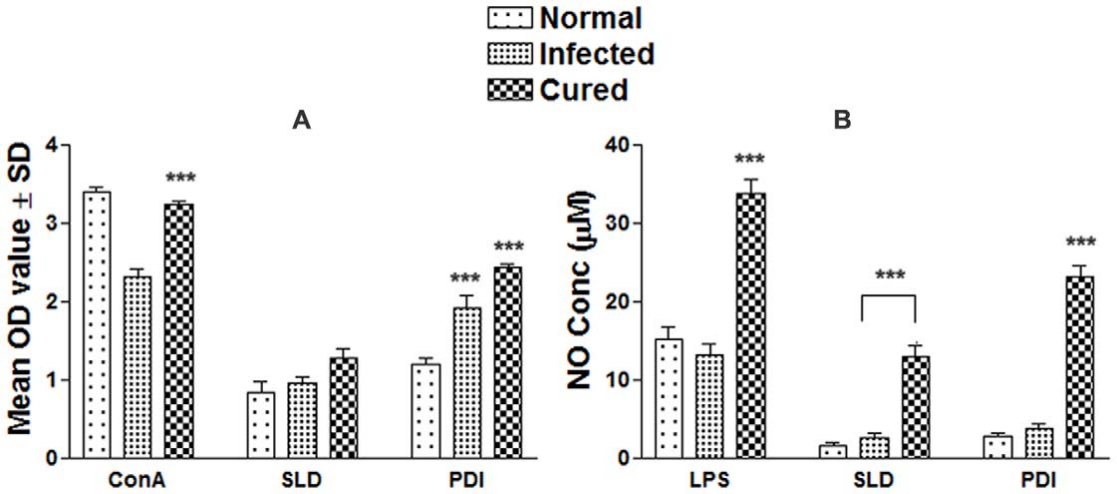
Cellular responses of rLdPDI of *L. donovani* in hamsters. (A) LTT response of mononuclear cells (lymph nodes) from normal, *L. donovani* infected (30 day p.c.) and treated hamsters in response to Con A, SLD and rLdPDI at 10 µg/ml each. Proliferation was represented as mean OD of stimulated culture - mean OD of unstimulated control. Each bar represents the pooled data (mean ± S.D. value) of 6 hamsters and the data represent the means of triplicate wells ± the S.D of each hamster. (B) Nitric oxide production (µM) by peritoneal macrophages of hamsters (n = 5). The peritoneal macrophages were primed with the supernatants of stimulated lymphocytes (3 days with mitogen and 5 days with antigens) of normal, infected and cured hamsters in response to rLdPDI, SLD and LPS respectively at10 µg/ml each. The estimation of NO production was done using Greiss reagent in supernatants collected from macrophage cultures 24 h after incubation and the absorbance of the reaction product was measured at 540 nm. Significance values indicate the stimulation of lymphocytes difference between the infected and cured in response to rLdPDI and compared with SLD (*, p<0.05; **, p<0.01; and ***, p<0.001).

The cellular responses of lymph node cells of cured hamsters were assessed by XTT against the Con A (mitogen) and SLD as well as rLdPDI antigens. The responses were compared with that of normal as well as *L. donovani* infected groups that served as controls. The normal control as well as cured *Leishmania* infected group had shown significantly higher proliferative responses against Con A as compared to *L. donovani*-infected group. The results of the proliferative response of lymphocytes against rLdPDI showed significantly higher stimulation in cured/infected hamsters (mean OD 2.437±0.11 and 1.983±0.29) than SLD (mean OD 1.27±0.28 and 0.968±0.14). The difference was statistically significant (p<0.001) (Fig. 2 A).

NO-mediated macrophage effector mechanism is known to be critical in the control of parasite replication in the animal model hence its production in peritoneal macrophages of cured hamsters, was studied after 24 h of incubation in the presence of rLdPDI and SLD. For comparison, NO production in mitogen (LPS) stimulated and unstimulated cells served as positive and negative controls respectively. NO production was recorded to be significantly higher against rLdPDI (p<0.001) (Fig. 2 B).

### rLdPDI stimulates PBMCs from Leishmania infected/cured/endemic to proliferate and to express a predominant Thl Cytokine Profile

**Figure 3:**
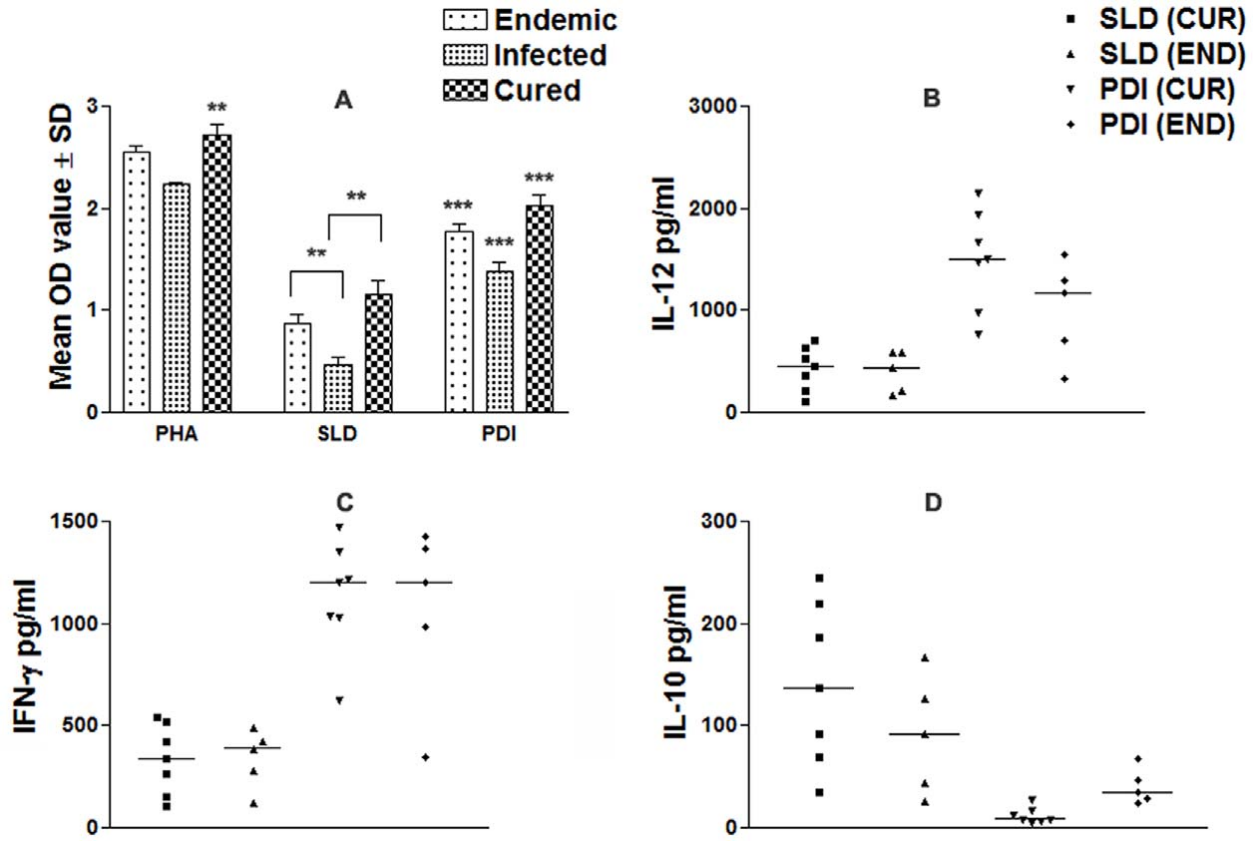
Cellular responses of rLdPDI of *L. donovani* in human PBMCs. LTT response of PBMCs in non endemic normal/healthy individuals, endemic contacts, leishmania infected patients and cured individuals. Each bar represent the mean OD ± SD value of stimulated PBMCs of each group (A); Th1 and Th2 cytokine production (B–D) in PBMCs from individuals of cured VL patients (7) and endemic controls (5) in response to rLdPDI and SLD antigens, each data point represents one individual. Values are given as concentration in pg/ml. The statistical significances are given between infected vs cured and infected vs endemic individuals. (*, p<0.05; **, p<0.01; and ***, p<0.001).

We further analyzed PBMCs of cured patients, endemic and non-endemic controls and *L. donovani*-infected donors for cellular and cytokine responses. The cytokine responses in individual donors in each study group were found to elicit different responses. Proliferation and cytokine responses of PBMCs from patients with active VL/cured/endemic were compared using rLdPDI and SLD. Endemic control and cured patients exhibited relatively higher mean OD values against PHA, i.e. 2.55±0.027 and 2.74±0.042 respectively. PBMCs from all the cured and active VL patients proliferated in response to rLdPDI with mean OD values of 2.33±0.037 and 1.52±0.052 higher values than SLD (mean OD values of 1.18±0.081 and 0.467±0.189) respectively (p<0.001) (Fig. 3 A). The results demonstrate that rLdPDI is a potent T cell antigen recognized by a majority of active VL/cured/endemic individuals in different stages or manifestations of infection.

To assess the Th1/Th2 stimulatory potential of the rLdPDI, we further estimated the cytokine levels viz. IFN-γ, IL-12 and IL-10 in PBMCs from cured/infected patients as well as in endemic and non endemic contacts against rLdPDI. The levels of IFN-γ and IL-12 were observed to be higher in the supernatants of cured patients with a range of 623–1470 pg/ml and 773–2145 pg/ml respectively as compared to endemic contacts (344–1427 pg/ml and 332–1553 pg/ml respectively). On the contrary, very low levels of IL-10 cytokines against rLdPDI were detected in supernatants of cured (17.15–40.12 pg/ml) patients followed by endemic contacts (ranging from 15.10–35.48 pg/ml). PBMCs of cured/endemic contacts generated a mixed Th1/Th2 cytokine profile against SLD, wherein high levels of IL-10, very little level of IFN-γ and IL-12p40 were noticed in response to SLD in cured/endemic contacts (Fig. 3 B–D). We were not able to detect these cytokines against rLdPDI in infected and non endemic individuals (data not shown).

### Serum of cured VL patients exhibited high level of IgG2 against rLdPDI

**Figure 4:**
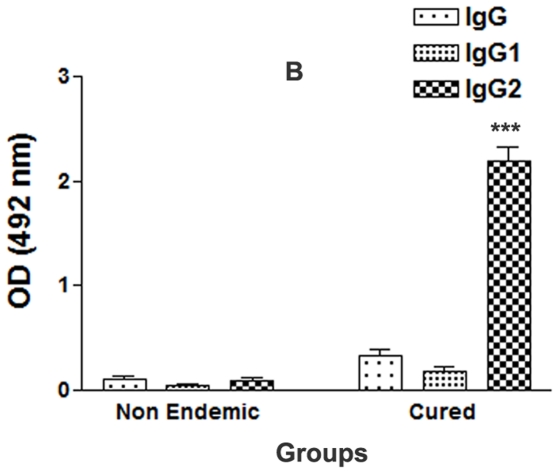
Antibody levels (OD value) in cured patients and non endemic individuals (6 each) against rLdPDI. Each bar represents the pooled data (mean±SD value) of three replicates. Significance values indicate the difference between the immunized group and normal group (*, p<0.05; **, p<0.01; and ***, p<0.001).

The estimation of the level of antibody- IgG and its isotypes in the serum of cured patients revealed a higher IgG2 response (mean OD, 2.192±0.135 and 0.392±0.12) than non endemic patients(mean OD, 0.087±0.025) (Fig. 4). The difference was statistically significant (p<0.001). However, the IgG1 response was not significantly different between the cured and the non endemic individuals (mean OD, 0.33±0.002 versus 0.29±0.015; p>0.5). The IgG2 level in cured patients serum was found to be >10 fold higher than non endemic individuals.

### Vaccination with pcDNA-*LdPDI* generates optimum protection in hamsters against *L. donovani*-challenges

Encouraged with the ability of rLdPDI to generate a Th1 type profile, we further evaluated the prophylactic efficacy of pcDNA-*LdPDI* construct in hamsters against *L. donovani* challenges. The pcDNA-*LdPDI* vaccinated hamsters were found to be optimally protected (∼90%) from the *L. donovani* challenge. No protective effect was seen when hamsters were immunized with the control vector.

**Figure 5:**
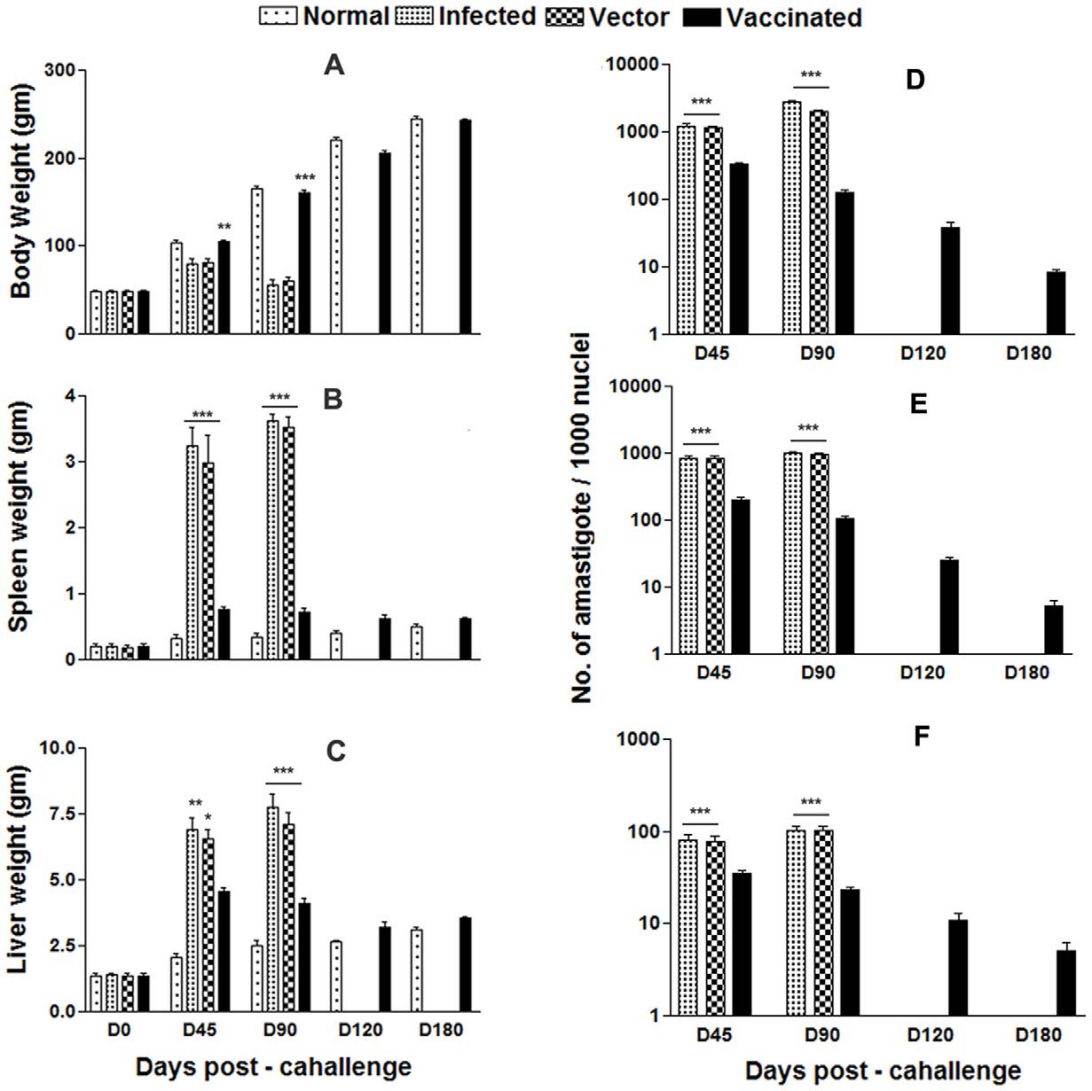
Body weight (A), spleen weight (B) and liver weight (C) in gm of normal, infected control, vector (pcDNA) control as well as pcDNA-*LdPDI* vaccinated hamsters. Parasite burden (number of amastigotes per 1000 cell nuclei) in the spleen (D), liver (E), and bone marrow (F) of infected control, vector (pcDNA) control as well as pcDNA-*LdPDI* vaccinated hamsters on days 0, 45, 90, 120, and 180 p.c.. Significance values indicate the difference between the vaccinated groups and infected group (*, p<0.05; **, p<0.01; and ***, p<0.001).

The vaccinated animals gained weight with time similarly as in normal animals whereas there was significant weight loss (p<0.001) in the animals of the infected and vector control groups (Fig. 5A). There was an absence of hepatosplenomegaly in the vaccinated group that is normally associated with the challenge infection (Fig. 5 B&C). An increase from ∼10^2^ to ∼10^4^ parasites in all of the groups, except in the pcDNA-*LdPDI* -vaccinated group, was seen in Giemsa-stained splenic smears from days 45 to 90 p.c. (Fig. 5 D–F). In the vaccinated group, parasite loads decreased from 2×10^2^ on day 45 to a very low level (p<0.001) by day 120 p.c. Similarly, in liver and bone marrow, parasite loads decreased sharply after day 45 p.c. and parasites were almost absent by day 180 p.c. in the same vaccinated group. Cultivation of the spleen, liver, and lymph node tissues from the vaccinated hamsters in *vitro* yielded no promastigotes after prolonged incubation for 3 wk.

The pcDNA-*LdPDI* -vaccinated hamsters survived the challenges of *L.donovani* and remained healthy more than 8 months. In contrast, hamsters vaccinated with the pcDNA3 vector and infected control survived for only 2–3 months.

### pcDNA-*LdPDI* vaccination stimulates DTH, mitogenic and Leishmania-specific cellular responses

DTH, an index of cell mediated immunity *in vivo*, and an antigen specific *in vitro* T cell proliferation assay revealed the status of cellular responses generated in vaccinated animals. We were therefore interested to see the DTH and proliferative responses elicited by vaccinated and challenged animals.

**Figure 6:**
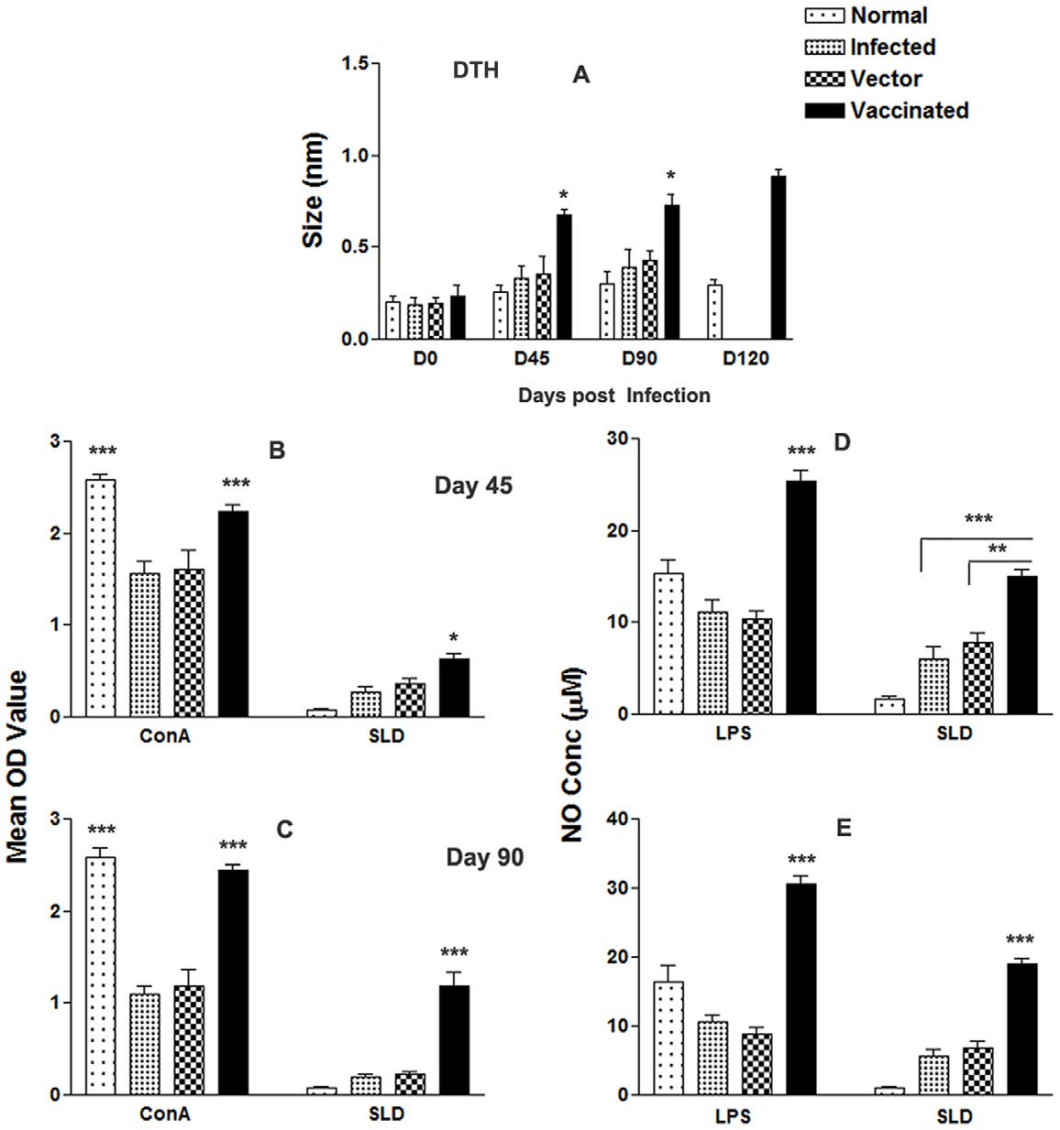
Cellular response against SLD in normal, infected control, vector (pcDNA) control as well as pcDNA-*LdPDI* vaccinated hamsters at different time intervals post challenge. (A), DTH response (mm). (B–C), LTT response (mean OD value) to SLD and Con A on days 45 (B–C) and 90 post challenge. (D–E), NO production (µM) to LPS and SLD in the naive macrophages coincubated with supernatants of lymphocytes isolated from pcDNA-*LdPDI* -vaccinated hamsters in comparison to the unimmunized infected controls, vector-immunized controls, and uninfected normal hamsters on day 45 and day 90 post challenge. Significance values indicate the difference between the vaccinated groups and infected group (*, p<0.05; **, p<0.01; and ***, p<0.001).

pcDNA-*LdPDI* -vaccinated hamsters displayed significant DTH responses, which increased progressively and was higher than those of the control groups (p<0.05) at all time points for the duration of the experiments for up to 120 days (Fig. 6 A). *In vitro* stimulation of the lymphocytes with Con A showed comparable proliferative responses at high levels in all of the groups when assayed before challenges. Cells from pcDNA-*LdPDI* -vaccinated hamsters produced a significantly higher response (p<0.001), which reached almost to the maximum on day 90 (Fig 6 B&C) as compared to infected and vector control against SLD.

NO is the critical killing effector molecule against leishmaniasis produced by IFN-γ stimulated and inducible NO synthase induced classical macrophages. Lymphocyte-mediated activation of macrophages to produce NO for leishmanicidal activities was found to differ between control and experimental groups of hamsters. Supernatants from stimulated lymphocytes of hamsters immunized with the pcDNA-*LdPDI*, when incubated with naive macrophages produced significant (p<0.001) amounts of nitrite (18 µM) which was ∼2 fold more than that of unimmunized infected and vector controls and ∼4-fold more than the normal control group on day 90 p.c.Fig. 6 D&E).

### pcDNA-*LdPDI* vaccination generates Th1-type cytokine profile as determined by quantitative real-time PCR

It is well established that the cytokine milieu at the initiation of infection is critical in determining disease outcome [45], [46]. So to understand the interplay between the disease healing inflammatory cytokines, IFN-γ and IL-12 as well as disease associated cytokines, IL-10 and IL-4, we sought to investigate the expression of these cytokines and the level of iNOS transcript, which play major role in leishmaniasis control, in infected control as well as vaccinated groups by qRT-PCR.

**Figure 7:**
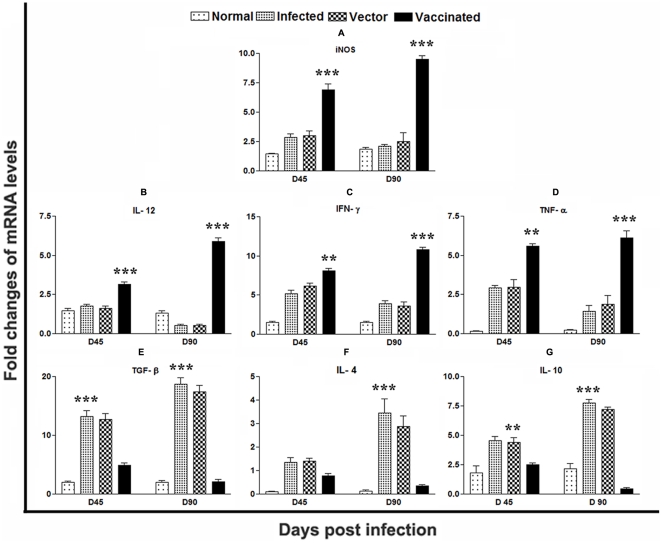
Splenic iNOS and Th1/Th2 cytokine mRNA expression profile analysis of normal and vaccinated hamsters on days 45 and 90 p.c. by quantitative real-time –PCR. Significance values indicate the difference between the vaccinated group and infected group (*, p<0.05; **, p<0.01; and ***, p<0.001).

The expression of iNOS transcripts was observed to be significantly elevated (p<0.001) in pcDNA-*LdPDI* vaccinated hamsters on day 45 by >2 fold and found to be increased by ∼4 fold at D 90 p.c. (Fig. 7 A). Its level was much lower in vector and infected control groups. Similar was the case with IL-12 which was least expressed in the infected group on day 90 p.c, but was significantly expressed by ∼5 fold (p<0.001) in vaccinated hamsters on days 90 p.c. (Fig. 7 B). The expression of IFN-γ, although variable at different time points, was suppressed in the infected group on day 90.p.c, but was significantly higher in the vaccinated group on this time point (p<0.001) (Fig. 7 C). The expression of TNF-α was also significantly higher by ∼6 fold (p<0.05) in the vaccinated group in comparison to *L. donovani*-infected group (Fig. 7 D). TGF-β mRNA expression was clearly up-regulated in the vector and infected groups. In contrast, mRNA expression for this cytokine was drastically suppressed in vaccinated group (Fig. 7 E). The level of IL-4, an established Th2 cytokine, was found to be suppressed in the vaccinated hamsters group and the level of Th1 suppressive cytokine IL-10 was noticed significantly higher in the vector and infected control groups as compared to the vaccinated group (Fig. 7 F&G).

### pcDNA-*LdPDI* vaccination alters Leishmania-specific IgG and its isotypes

**Figure 8:**
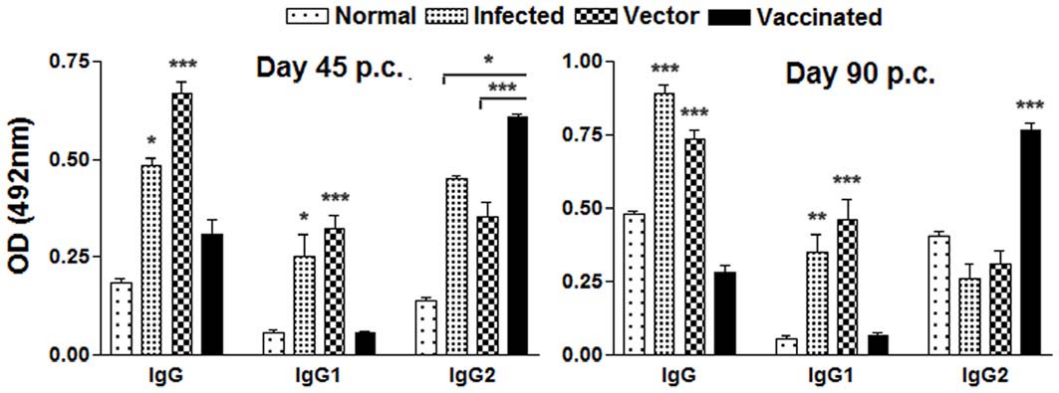
Anti- leishmania-specific IgG and its isotypes IgG1 and IgG2 in pcDNA-*LdPDI* -vaccinated hamsters in comparison to the unimmunized infected hamsters on days 45 and 90 p. c.. Significance values indicate the difference between the vaccinated group and infected group (*, p<0.05; **, p<0.01; and ***, p<0.001).

The serum levels of leishmanial antigen-specific IgG and its isotypes (IgG1 and IgG2) from all of the groups were assessed by ELISA. The anti-Leishmania IgG and IgG1 were elevated progressively with time to a high level in all the experimental groups, except the pcDNA-*LdPDI* vaccinated, in which case they remained essentially the background levels of the infected and vector immunized groups. In contrast, pcDNA-*LdPDI* vaccinated animals were the only group that showed a significant elevation by 2- to 3 fold over the others (p<0.001) in the level of IgG2 (Fig. 8). As a measure of CMI, the elevation of IgG2 was consistent with the development of effective immune responses. These results are in synchrony with the high level of IFN-γ, as well as IL-12 cytokine responses and suggest that the CD4+ T-cell response elicited by the pcDNA-*LdPDI* vaccine is preferentially of the Th1 phenotype.

## Discussion

In Leishmania species, Protein disulfide isomerase (PDI) - a redox chaperone, is reported to be involved in its virulence and survival. Several studies have described the virulence factors as potentially immunogenic in humans as well as in rodents (mice) and dogs [47], [48], [49].

PDI has been identified as a Th1 stimulatory protein from a sub-fraction of soluble Leishmania antigen through proteomics, belonging to the molecular weight range of 89.9 to 97.1 kDa [50]. Meek et al. [51] have detected IgG specific to four LaPDIs (especially to the 52-kDa PDI). Further, Ben Achour et al. 2002 reported that the 52-kDa PDI is linked to *L. major* virulence [35] and that there is a correlation between B cell activation/Ab production and lesion progression in *Leishmania*-infected patients and experimental animals [52], [53]. Padilla et al. [54] reported a unique PDI of ∼15 kDa, which was found to be essential for survival of *L. donovani*. In the present study, we have cloned *LdPDI* which exhibited very close homology with *L. infantum* PDI to the tune of 99%. Immunoblot study of *L. donovani* promastigotes lysate with the polyclonal anti-rLdPDI antibody has revealed one dominant protein of ∼55 kDa. This protein was identified earlier at high molecular weight range in proteomic studies which is in contrast to its observed molecular mass. This could be attributed to the post-translational modifications which are widely prevalent in Leishmania [30], [55].

Further, to evolve its function, LdPDI was expressed in the *E. coli* Rosetta strain with pET28a vector and in HEK mammalian cells (at the RNA and protein levels using the pcDNA3 vector). The successful delivery and the expression of the cloned *LdPDI* in the HEK cell line further authenticate its evaluation as a DNA vaccine candidate.

The protein was further characterized for its immunogenicity *in vitro*. We have reported earlier that a T cell response develops when cells from the cured patients from *Leishmania* endemic regions are stimulated with SLD and its fractions and subfractions [37], [56]. rLdPDI, being one of the Th1 stimulatory protein identified from the subfraction of SLD in the range of 89.9 to 97.1 kDa, when was subjected to re-evaluation for its stimulatory potential in *Leishmania* infected and cured hamsters, induced better cellular immune response as compared to SLD. Its immunogenicity was further validated successfully in endemic non-immune donors (household contacts without any clinical symptoms) and in immune patients of VL that were cured either with amphotericin B or miltefosine.

Due to the existence of dichotomy in the human system, there has been reservation in accepting human Th1 and Th2 to the rodent Th1 and Th2 subsets. Moreover, in murine VL, while the self-limiting infection pattern does not simulate the human profile, the systemic infection of the hamster with *L. donovani* is very similar to human Kala- azar, as it results in a relentlessly increasing visceral parasite burden, progressive cachexia, hepatosplenomegaly, pancytopenia, hypergammaglobulinemia, and, ultimately, death [36]. Hence, analysis of cellular immune response of the rLdPDI was carried out using hamsters' lymphocytes/macrophages in order to correlate the observations made with the human lymphocytes. We have previously shown that the cellular responses to the fractions and subfractions of *L. donovani* promastigotes were similar in endemic controls/cured patients of VL, as well as in hamsters, indicating that the results so obtained with the hamster could be translated into humans [37], [56].

In the absence of cytokine reagents against hamsters, in this study we have observed the effect of rLdPDI on lymphocyte proliferation and NO production by peritoneal macrophages of hamster. It is well documented that in case of leishmanial infections, macrophages become activated by IFN-γ released from parasite-specific T cells, and are able to destroy intracellular parasites through the production of several mediators, principal among which is NO [57], [58]. rLdPDI gave significantly higher cellular responses viz. lymphoproliferative as well as NO release against all the cured hamsters in comparison to normal and infected ones.

Some of the recombinant antigens have previously been shown to induce lymphocyte proliferation and IFN-γ production in subjects cured of visceral and in patients with cutaneous or mucosal leishmaniasis [59], [60]. Since recovery from *Leishmania* infection, relies on the induction of the Th1 response [61], [62] with production of IFN-γ and IL-12 and enhanced expression of NO synthase [57], [63], in this study also we have observed that in response to rLdPDI, there was a release of very high amount of IFN-γ and IL-12p40, in endemic control and cured patients of VL and suppression of IL-10 as well. However, the overall proliferative/cytokine response of T-cells of endemic controls was not as high as was observed in recovered ones. The observation that rLdPDI stimulated T cells from cured/endemic individuals to proliferate and produce IFN-γ suggests that PBMCs' response to rLdPDI may be associated with protective immunity. We have also detected very high level of IgG2, a marker for Th1 response, in cured patients' serum against rLdPDI, reflecting its Th1 stimulatory potential.

Encouraged with these observations we further evaluated the prophylactic potential of LdPDI as a plasmid DNA vaccine to *L. donovani* challenges. It has been shown in a variety of experimental models of infection that vaccination with plasmid DNA induces protective immunity through both MHC class I- and class II-restricted T cell responses [24]. On the other hand, vaccination with conventional protein, in general, induces MHC class II- but not class I-restricted responses. For those infections in which IFN-γ and/or CTL responses may be required, the ability of DNA vaccination to elicit an MHC class I response may be advantageous over conventional protein vaccination in providing a more broad-based and potentially durable immune response. On the basis of this view, the naive hamsters were vaccinated with pcDNA-*LdPDI* and challenged with the virulent strain of *L. donovani.* Interestingly, the immunized hamsters survived the lethal challenge and remained healthy after the termination of the experiment at day 180 p.c., whereas all non-immunized and blank vector-immunized hamsters succumbed to the lethal *L. donovani* challenge within 3–4 months p.c.

A major factor of the immune mechanism(s) is the development of strong cell-mediated immunological (CMI) responses like T-cell responses, NO production and DTH responses which are responsible for protection and are also supposed to contribute to healing in VL [29], [64], [65], [66]. In the present study, all the pcDNA-*LdPDI* - vaccinated hamsters challenged with *L. donovani* have shown a specific active T-cell response because they displayed significant lympho-proliferative responses after challenge as compared to non-immunized infected and healthy control hamsters where it was severely impeded. Further, the supernatant of SLD-stimulated lymphocytes from pcDNA-*LdPDI* -vaccinated hamsters produce a remarkable level of NO in the macrophages of naive hamsters which also support the view regarding the up-regulation of iNOS by Th1 cell-associated cytokines and confirms that the NO-mediated macrophage effector mechanism is critical in the control of parasite replication in the animal model [67]. Moreover, successful vaccination of humans and animals is often related to antigen-induced DTH responses *in vivo* and T-cell stimulation with antigen *in vitro* [36], [68], suggesting a correlation between cell mediated immune responses and immunity to infection in this model. Here too, a low level of parasite-specific DTH responses observed in infected and vector control animals can be correlated with disease progression in hamsters which, on the other hand, was strongly elevated in hamsters immunized with pcDNA-*LdPDI* vaccine. Apart from diminished cellular responses, active VL is also associated with the production of high levels of the *Leishmania* specific antibody, which is observed before detection of parasite-specific T cell response [69]. It has been well known that as a measure of CMI, the elevation of IgG2, which was significantly and consistently prominent in the vaccinated group of this study, is consistent with the development of effective immune responses [44] together with the progressive elevation of the anti-Leishmania IgG and IgG1 in all the groups except the vaccinated one, suggested that protection against leishmaniasis is induced by a strong T cell response [70], [71]. The presence of a comparable existence of Th1 and Th2 clones producing both IFN-γ and IL-4 obtained from patients cured of VL encouraged us to assess whether the protective response which was utmost elicited by pcDNA-*LdPDI* vaccination in hamsters can reflect this feature of clinical findings [72], [73], [74]. Notably, we have observed that vaccination with pcDNA-*LdPDI* resulted in significant elevation of Th1 type of immune response. The transcript of IFN-γ, a signature cytokine of the Th1-type response having a dominant effect on macrophage microbicidal responses and other effector killing mechanisms, along with TNF-α, often reported to act in concert to activate iNOS for the production of NO [75], [76], were found to be down-regulated [64] in infected hamsters, whereas their expression was observed to be increased many fold in the immunized hamsters. The key macrophage deactivating Th2 cytokines- IL-10 and IL-4 have a definitive association with an acute phase of VL during which their level increase progressively in tissues but were not detectable after successful cure [17], [36]. Commensurate with these observations, an extreme down-regulation of IL-10 and IL-4 mRNA levels was observed in pcDNA-*LdPDI* -vaccinated hamsters as compared with infected control hamsters [77]. Furthermore, the presence of IL-10 as well as IFN-γ was reported in patients with acute VL whereby only IL-10 levels decreased remarkably with disease cure [78]. In addition, there are reports that primary Th1 cell-mediated antileishmanial events induced in IL-10-deficient mice require IFN-γ that is largely induced by IL-12 [29], [79]. However, in the present study, IL-12 was completely down-regulated in infected hamster, whereas high level of IL-12 mRNA transcript was found in vaccinated hamsters. TGF-β, a pleiotropic cytokine with diverse functions, is known to be expressed at a moderate level even in normal hamsters, [29], [36], [64] was observed to be markedly upregulated in *L. donovani* infected hamsters but severely down regulated in vaccinated animals. Furthermore, there was apparent down-regulation of IL-4 expression in all of the immunized hamsters throughout the experiment. Finally, unlike mice where IL-4 and IL-12 direct IgG subclass switching of IgG1 and IgG2a, respectively, such distinct IgG classes remains obscure in hamsters [42], [80]. It has been well established that IgG and IgG1 antibody increase in titre with the *L. donovani* loads [29]. Therefore, the very low level of these antibodies is thus consistent with the decreasing parasite loads seen in the vaccinated group. The significant increase in the IgG2 levels only in vaccinated animals is indicative of enhanced CMI.

In summary, this study demonstrates for the first time the immunogenicity of rLdPDI protein both *in vitro* as well as *in vivo,* eliciting a dominant Thl-type cytokine profile. In addition, the significant prophylactic efficacy of the pcDNA-*LdPDI* makes it a strong and promising prophylactic vaccine candidate.
